# Inhibition of CCN6 (WISP3) expression promotes neoplastic progression and enhances the effects of insulin-like growth factor-1 on breast epithelial cells

**DOI:** 10.1186/bcr1351

**Published:** 2005-11-08

**Authors:** Yanhong Zhang, Quintin Pan, Hui Zhong, Sofia D Merajver, Celina G Kleer

**Affiliations:** 1 Department of Pathology, University of Michigan Medical Center, 1150 W Medical Center Drive, Ann Arbor, MI 48109, USA; 2 Department of Internal Medicine, Division of Hematology/Oncology, University of Michigan Medical Center, 1500 E. Medical Center Dr., Ann Arbor, MI 48109, USA; 3 Comprehensive Cancer and Geriatrics Center, University of Michigan Medical Center, 1500 E. Medical Center Dr. Ann Arbor, MI 48109, USA

## Abstract

**Introduction:**

CCN6/WISP3 belongs to the CCN (Cyr61, CTGF, Nov) family of genes that contains a conserved insulin-like growth factor (IGF) binding protein motif. CCN6 is a secreted protein lost in 80% of the aggressive inflammatory breast cancers, and can decrease mammary tumor growth *in vitro *and *in vivo*. We hypothesized that inhibition of CCN6 might result in the loss of a growth regulatory function that protects mammary epithelial cells from the tumorigenic effects of growth factors, particularly IGF-1.

**Method:**

We treated human mammary epithelial (HME) cells with a CCN6 hairpin short interfering RNA.

**Results:**

CCN6-deficient cells showed increased motility and invasiveness, and developed features of epithelial-mesenchymal transition (EMT). Inhibition of CCN6 expression promoted anchorage-independent growth of HME cells and rendered them more responsive to the growth effects of IGF-1, which was coupled with the increased phosphorylation of IGF-1 receptor and insulin receptor substrate-1 (IRS-1).

**Conclusion:**

Specific stable inhibition of CCN6 expression in HME cells induces EMT, promotes anchorage-independent growth, motility and invasiveness, and sensitizes mammary epithelial cells to the growth effects of IGF-1.

## Introduction

Wnt-1-induced secreted protein 3 (CCN6/WISP3) is a cysteine-rich protein that belongs to the CCN (Cyr61, CTGF, Nov) family of genes, which also contains five other members: CCN1 (connective tissue growth factor or CTGF), CCN2 (Cyr61), CCN3 (Nov), CCN4 (WISP1) and CCN5 (WISP2) [1-3]. CCN4, the first cloned member of the WISP proteins was discovered because it was upregulated in the mouse mammary epithelial cell line C57MG transformed by Wnt-1 [2]. CCN6 shares high sequence homology to CCN4, although there is no evidence that CCN6 is induced by Wnt-1 signaling [2]. CCN growth factors are mostly secreted and extracellular matrix-associated proteins that regulate important functions including cellular differentiation and development, cell proliferation, survival, angiogenesis, cell migration and adhesion [2-6]. CCN proteins interact with key signaling molecules such as cell surface integrins, NOTCH1, fibulin C, S100A4, and ion channels [7-12].

CCN proteins contain an N-terminal secretory signal, followed by four distinct motifs with homology to insulin-like growth factor (IGF)-binding protein; von Willebrand factor type C; thrombospondin 1 (TSP1); and a C-terminal region with heparin-binding motifs and sequence similarities to the C termini of von Willebrand factor and mucins, the latter also being present in other growth factors such as transforming growth factor-β, platelet-derived growth factor, and nerve growth factor [1,2,9]. It is becoming increasingly evident that CCN proteins function in a cell-type-specific and tissue-type-specific manner. For example, increased expression of CCN3 (Nov) was correlated with reduced tumorigenicity in glioma, choriocarcinoma, and Ewing's sarcoma cell lines [13-16]. However, increased expression of CCN3 was associated with enhanced tumor growth in renal cell carcinomas [17]. CCN2 (Cyr61) stimulates the growth of breast and gastric adenocarcinomas [18,19], whereas its expression is downregulated in non-small cell lung cancer, in which CCN2 suppressed the growth of NSCLC cells by triggering a signal transduction pathway through β-catenin [20,21]. These data suggest that two members of the CCN family, CCN2 and CCN3, behave under certain circumstances as oncogenes or tumor suppressor genes in different tissue types (reviewed in [12,22]).

CCN6 maps to chromosome 6q21-22, a locus that displays high rates of loss of heterozygosity in breast cancer [23,24]. We have previously reported that CCN6 is lost in the majority of inflammatory breast cancers, a highly aggressive and metastatic form of breast cancer [25]. Restoration of CCN6 expression in inflammatory breast cancer cells results in growth inhibition *in vitro *and *in vivo *[6,26]. After synthesis, CCN6 protein is secreted and is able to modulate the effects of IGF-1 on IGF-1 receptor (IGF-1R) activation and its signaling pathways [27]. Here we investigate the function of CCN6 in the normal breast epithelium. We show that inhibition of CCN6 expression in human mammary epithelial cells strongly induces epithelial-mesenchymal transition (EMT) and leads to a highly motile and invasive phenotype. CCN6 loss promotes anchorage-independent growth in breast epithelial cells and increases cell proliferation, which is mechanistically driven, at least in part, by enhancement of the growth effects of IGF-1 and the activation of IGF-1R signaling pathways.

## Materials and methods

### Cell culture

Human mammary epithelial (HME) cells were immortalized with human papilloma virus E6/E7 and were characterized as being positive for keratin 19 [6,28]. Cells were cultured in Ham's F-12 medium supplemented with 5% fetal bovine serum (FBS), hydrocortisone (1 μg/ml), insulin (5 μg/ml), epidermal growth factor (EGF; 10 ng/ml), cholera toxin (100 μg/ml), fungizone (2.5 μg/ml) and gentamycin (5 μg/ml), at 37°C under 10% CO_2_.

### Generation of stable hairpin short interfering RNA-CCN6 HME cells

Hairpin short interfering RNA (siRNA) 5'-GATCCCGCCAGGGGAAATCTGCAATGTTCAAGAGACATTGCAGATTTCCCCTGGTTTTTTGGAAA-3' and the complementary strand 5'-AGCTTTTCCAAAAAACCAGGGGAAATCTGCAATGTCTCTTGAACATTGCAGATTTCCCCTGGCGG-3' were synthesized (Invitrogen, Carlsbad, CA, USA), and annealed hairpin siRNA-CCN6 inserts were cloned into pSilencer2.1-U6 hygro expression vector (Ambion, Austin, TX, USA). The sequence of siRNA-CCN6 (insert in) expression vector was confirmed by sequencing (University of Michigan DNA Sequencing Core). HME cells were transfected with pSilencer2.1-U6 negative control (siRNA-control; Ambion) or siRNA-CCN6 plasmids by using FuGene 6 transfection reagent (Roche-Boehringer Mannheim, Mannheim, Germany). Stable transfectants were established by culturing transfected cells in the described medium supplemented with 100 μg/ml hygromycin (Invitrogen) for 4 weeks.

### RT-PCR analysis

Total RNA was isolated from HME cells and siRNA HME clones with a Trizol kit (Life Technologies, Inc., Gaithersburg, MD, USA). First-strand cDNA synthesis was performed by using 1 μg of total RNA with AMV reverse transcriptase (Promega, Madison, WI, USA) and oligo(dT) as a primer. A 2 μl portion of the reaction mixture was used for amplification by PCR. The following primers were used: CCN6 forward primer 5'-ATGCAGGGGCTCCTCTTCTCC-3' and CCN6 reverse primer 5'-CTTGAGCTCAGAAAATATATC-3' to amplify a 1,050 bp product; E-cadherin forward primer 5'-CCTTCCTCCCAATACATCTCCC-3' and E-cadherin reverse primer 5'-TCTCCGCCTCCTTCTTCATC-3' to amplify a 600 bp product. PCR was performed under the following conditions: denaturing for 1 minute at 94°C, annealing for 1 minute at 58°C for CCN6 and at 65°C for E-cadherin, and elongation for 1 minute at 72°C, for 35 cycles.

### Immunofluorescence

Cells were grown on glass coverslips, fixed with methanol for 5 minutes at -20°C and then permeabilized with 0.2% Triton X-100 for 15 minutes at room temperature 25°C. The coverslips were then saturated for 30 minutes with 2% bovine serum albumin in PBS. After being washed in PBS, cells were incubated for 1 hour with a monoclonal antibody against E-cadherin (dilution 1:50, BD Transduction Laboratories, San Jose, CA, USA). Cells were exposed for 1 hour to Alexa Fluor anti-mouse Ig G (Molecular Probes, Eugene, OR, USA). After being washed with PBS three times, the coverslips were then mounted with prolong Gold anti-fade reagent with 4',6-diamidino-2-phenylindole and the borders were sealed with transparent nail polish.

### Anchorage-independent growth

For studies of anchorage-independent growth we performed soft agar assays on stable clones of HME/CCN6 siRNA and on HME control cells. Each well of a six-well plate was first layered with 0.6% agar diluted with 5% FBS-supplemented Ham's F-12 medium complete with growth factors. The cell layer was then prepared by diluting agarose to concentrations of 0.3% with 5 × 10^3 ^cells in 2.5% FBS-supplemented Ham's F-12 (1.5 ml per well). Plates were maintained for 3 weeks at 37°C under 10% CO_2_. Colonies were counted under a microscope with a grid. The experiment was performed three times independently.

### Basement membrane matrix invasion

HME/CCN6 siRNA and HME controls were trypsinized and 300 μl of 10^6 ^cells/ml in serum-free medium were seeded at equal numbers onto the extracellular matrix layer (ECM; Chemicon, Temecula, CA, USA). Subsequently, we added 500 μl of medium containing 5% FBS to the lower chamber. After incubation for 24 hours, the non-invading cells and ECM gel from the interior of the insert were removed gently with a cotton swab. The invasive cells on the lower surface of membrane were stained, air dried, and photographed. The invaded cells were counted under the microscope. The experiment was performed three times independently.

### Random motility

Random cell motility was determined as described in the motility assay kit (Cellomics Inc., Pittsburg, PA, USA). Cells were harvested and 500 cells per well were suspended in 2.5% FBS medium and plated on top of a field of microscopic fluorescent beads in a 96-well plate. After incubation for 16 hours, cells were fixed and areas of clearing in the fluorescent bead field corresponding to phagokinetic cell tracks were quantified with an NIH ScionImager. The experiment was performed three times independently.

### Immunoprecipitation

HME cells and HME/CCN6 siRNA cells were serum-starved for 24 hours and subsequently stimulated for 15 minutes with 25 ng/ml IGF-1. Proteins were obtained by lysing the cells in a buffer composed of 50 mM Tris-HCl, pH 7.4, 150 mM NaCl, 1% Nonidet P40, 1 mM Na_3_VO_4_, 1 mM phenylmethylsulphonyl fluoride, 1 μg/ml leupeptin, and 10 μg/ml aprotinin. The IGF-1R was immunoprecipitated overnight from 500 μg of cell (protein) lysate with anti-IGF-1R monoclonal antibody (Oncogene, San Diego, CA, USA) at 4°C. After isolation with protein A/G PLUS-Agarose (Santa Cruz Biotechnology, Santa Cruz, CA, USA), the immunocomplexes were separated by 7.5% SDS-PAGE. The proteins were transferred to a poly(vinylidene difluoride) membrane and subsequently detected by immunoblotting with anti-IGF-1R β subunit polyclonal antibody (Santa Cruz Biotechnology). Tyrosine phosphorylation of immunoprecipitated IGF-1R was assessed with anti-phosphotyrosine monoclonal antibody PY20 (Transduction Laboratories, Lexington, KY, USA).

### Western blot analysis

Cells were lysed in lysis buffer (50 mM Tris-HCl, pH 7.4, 150 mM NaCl, 1% Nonidet P40, 1 mM Na_3_VO_4, _1 mM phenylmethylsulphonyl fluoride, 1 μg/ml leupeptin, and 10 μg/ml aprotinin). Cell lysates (50 μg of protein) were fractionated by SDS-PAGE and transferred to Immobilon-P membrane (Millipore, Billerica, MA, USA). The membranes were blocked for 1 hour with Tris-buffered saline containing 5% non-fat milk and 0.1% Tween 20. Immobilized proteins were probed with antibodies specific for phosphorylated and total insulin receptor substrate-1 (IRS-1; Upstate Biotechnology), E-cadherin and vimentin monoclonal antibodies (BD Transduction Laboratories) and antibodies against cytokeratin 18 (c-04, ab668, Abcam Inc, Cambridge MA, USA). CCN6 was detected with a polyclonal anti-CCN6 antibody developed against an immunogenic peptide Ac-PEGRPGEVSDAPQRKQ-CONH_2 _corresponding to amino acids 31 to 46 of human CCN6 with the assistance of Covance.

### IGF-1 growth assay

HME/CCN6 siRNA and HME control cells were plated in 96-well plates at a concentration of 5 × 10^4 ^cells/ml and serum-starved for 24 hours. Subsequently, 25 ng/ml human recombinant IGF-1 (Upstate Biotechnology) was added. 3-(4,5-Dimethylthiazol-2-yl)-2,5-diphenyl-2*H*-tetrazolium bromide (MTT) reagents were added 24 hours later in accordance with the manufacturer's protocol, and the plate was read at a wavelength of 595 nm. The experiment was performed with triplicate samples.

### S-phase analysis

HME/CCN6 siRNA and HME control cells were plated at a concentration of 5 × 10^4 ^cells/ml and serum-starved for 24 hours. Subsequently, 25 ng/ml human recombinant IGF-1 (Upstate Biotechnology) was added. Serum-starved and IGF-1-treated HME control cells and HME/CCN6 siRNA cells were pulsed for 2 hours with 10 μM bromodeoxyuridine (BrdU; Roche, Indianapolis, IN, USA), harvested with trypsin-EDTA, and fixed with 70% ethanol at -20°C for at least 1 hour. Cells were resuspended in 2 M HCl for 20 minutes at room temperature. After centrifugation, the cell pellets were resuspended in borax (pH 8.5) to neutralize any residual acid. After brief centrifugation the pellets were washed in 1 ml PBS and resuspended for 1 hour in fluorescein isothiocyanate-labeled anti-BrdU antibody (catalogue no. 347583; Becton Dickinson San Jose, CA, USA) in the dark. Cells were washed in PBS, resuspended in PBS containing propidium iodide, and analyzed for propidium iodide and BrdU staining on a Becton-Dickinson flow cytometer with the use of Cellquest software. The experiment was performed three times independently.

## Results

### Expression of CCN6 in immortalized human mammary epithelial cells

A CCN6-specific receptor has not yet been cloned; blockade of the receptor is therefore not yet feasible as a means of inhibiting CCN6 function. Thus, to investigate the role of CCN6 in the mammary epithelium and in breast tumorigenesis, HME cells were treated with a CCN6 hairpin siRNA. siRNAs silence gene expression in a sequence-specific post-translational way; they therefore constitute a useful strategy for identifying gene function [29].

**Figure 1:**
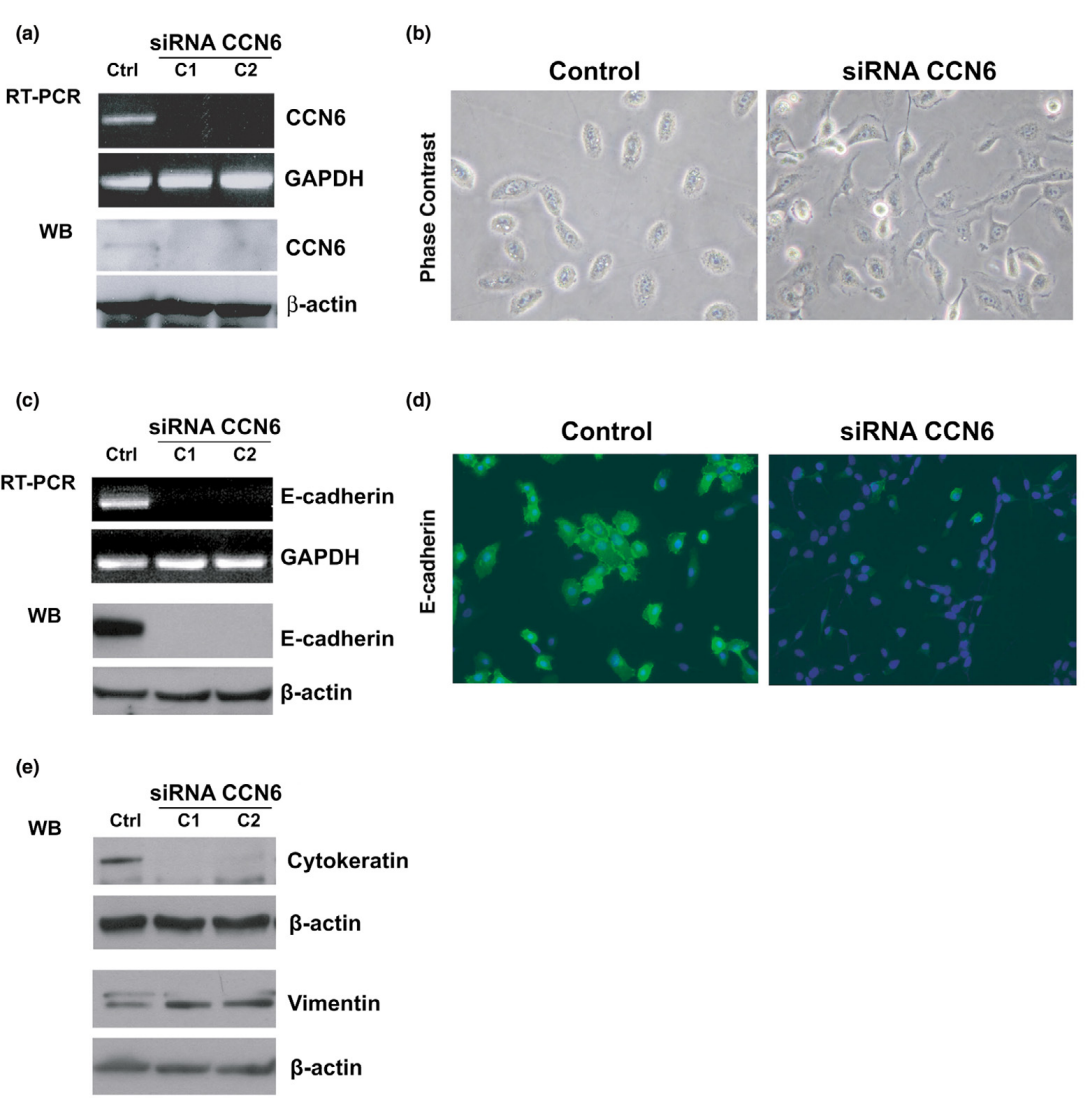
Effect of CCN6 inhibition on the phenotype of mammary epithelial cells. **(a) **Human mammary epithelial (HME) cells were stably transfected with pSilencer2.1-U6 hygro expression vector expressing CCN6 hairpin siRNA. The suppression of CCN6 was confirmed by reverse transcriptase PCR and Western immunoblotting (WB). HME control cells express low levels of CCN6 protein. C1 and C2 (two stable HME CCN6 siRNA clones) show almost complete inhibition of CCN6. **(b) **Stable inhibition of CCN6 on HME cells results in a change from compact and oval-shaped cells to irregularly shaped cells with many protruding processes. **(c) **E-cadherin protein and mRNA were decreased in CCN6-deficient cells. **(d) **By immunofluorescence CCN6-deficient cells show a drastic decrease of membrane-associated E-cadherin protein. **(e) **Western immunoblots show that CCN6 inhibition results in a decrease in cytokeratin and an increase in vimentin proteins. GAPDH, glyceraldehyde-3-phosphate dehydrogenase.

The expression of CCN6 transcript and protein in HME controls and HME cells stably transfected with CCN6 siRNA were studied by RT-PCR and Western immunoblotting, respectively (Fig. 1a). For subsequent experiments, two clones with CCN6 protein downregulation were selected to study the functional consequences of CCN6 inhibition. The hairpin inhibition of CCN6 protein levels was stable and remained stable 5 months after transfection, as tested by Western blotting.

### CCN6 inhibition is a potent inducer of EMT and triggers motile and invasive properties on mammary epithelial cells

While the parental HME cells were compact, thick, and ovoid, with rare extended processes, CCN6 inhibition led to cells that were thin, spreading, with stellate shape and numerous cytoplasmic extended processes reminiscent of an epithelial to mesenchymal transition (EMT) (Fig 1b). Consistently, CCN6-deficient cells had loss of the epithelial markers E-cadherin and cytokeratin, whereas they exhibited increased vimentin protein, a characteristic marker for mensenchymal cells (Fig. 1c,d and 1e).

**Figure 2:**
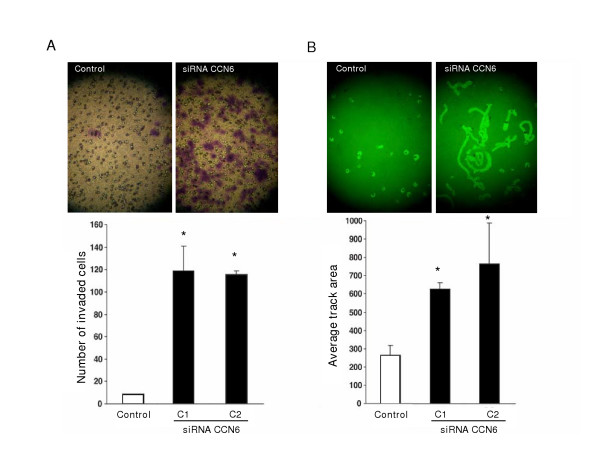
Effect of CCN6 inhibition on random motility and invasion. **(a) **A reconstituted basement membrane invasion chamber assay was used to investigate the effects of CCN6 inhibition on the invasion abilities of HME cells. Representative fields of invaded and stained cells are shown. *t* test *p < 0.04 vs. control. **(b) **Cell motility on a field of microscopic fluorescent beads. Representative photomicrographs show the clearing in the fluorescent bead field corresponding to phagokinetic cell tracks. *p < 0.005 vs. control. Experiments were performed independently three times. C1 and C2 are two stable CCN6 deficient clones.

Because E-cadherin is crucial for epithelial cell-cell adhesion and its downregulation has been shown to enhance cellular motility and invasion, we tracked the movement of CCN6-deficient cells and their ability to invade a basement membrane. As shown in Fig. 2, CCN6 deficient cells were much more motile and invasive *in vitro *than the controls.

### CCN6 inhibition promotes anchorage-independent growth, increases IGF-1R phosphorylation and potentiates the mitogenic effects of IGF-1

**Figure 3:**
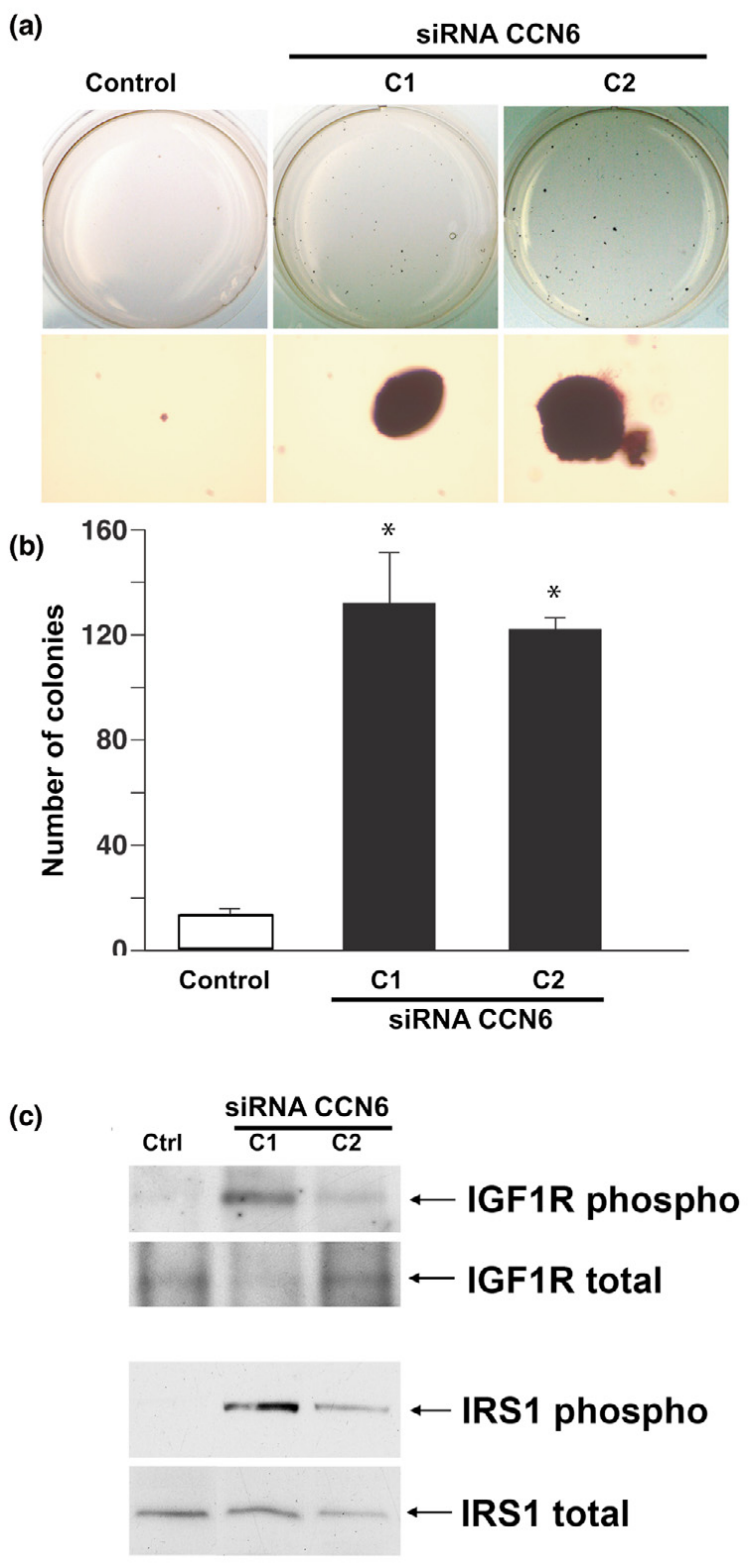
CCN6-deficient cells acquire anchorage-independent growth abilities. **(a) **Anchorage-independent growth of human mammary epithelial stable cell lines in soft agar. The upper panels show an assay in soft agar; the lower panels show the colony size under a × 100 magnification of the same wells after 3 weeks in culture. Three independent experiments yielded similar results. **(b) **Bar graph summarizing the results of three independent experiments in soft agar. **(c) **CCN6 inhibition induced an increase in phosphorylation of insulin-like growth factor-1 receptor (IGF-1R) and insulin receptor substrate-1 (IRS-1). C1 and C2 are two stable CCN6 deficient clones.

Stable inhibition of CCN6 by hairpin siRNA strongly promoted the growth of HME cells in soft agar (Fig. 3). CCN6-deficient cells were able to form colonies in medium supplemented with only 2.5% FBS, whereas HME control cells did not significantly form colonies under these conditions. CCN6-deficient cells formed about 10-fold more colonies than the control cells after 21 days. Furthermore, the colonies formed by the CCN6-deficient cells were much larger than the colonies formed by the control cells.

Our observation of the ability of CCN6-deficient cells to form colonies in soft agar in the presence of limiting serum suggested that these lines might have increased activation of growth receptor tyrosine kinase signaling pathways. This is a well-known mechanism for transformation that allows the cells to grow in the absence, or with minimal amounts, of growth factors [30,31]. Because CCN6 contains an IGFBP motif and once secreted it is able to modulate IGF signaling pathways [27], it was logical to begin our analysis by investigating whether inhibition of CCN6 might directly or indirectly induce the activation of IGF-1R. Stable CCN6 inhibition by hairpin siRNA led to an increase in the phosphorylation of IGF-1R and IRS-1 (Fig. 3c).

**Figure 4:**
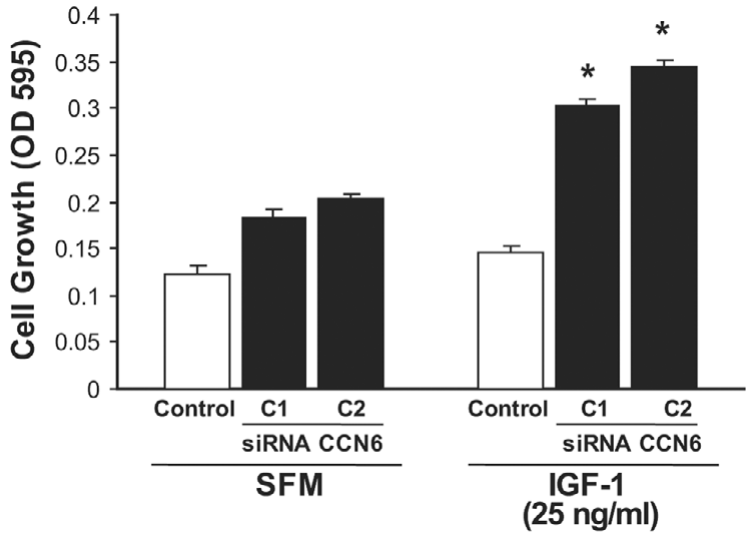
CCN6 inhibition increases the growth of human mammary epithelial cells in response to insulin-like growth factor-1 (IGF-1). Cells were serum starved for 24 hours, and subsequently stimulated with 25 ng/ml of IGF-1. MTT (3-(4,5-dimethylthiazol-2-yl)-2,5-diphenyl-2*H*-tetrazolium bromide) reagents were added 24 hours later and the plate was read at a wavelength of 595 nm. Results are expressed as means ± SD for three independent experiments. Asterisks indicate statistical significance (*t *test); *p *< 0.005 for short interfering RNA (siRNA)/CCN6 after treatment with IGF-1 compared with serum free medium (SFM). C1 and C2 are two stable CCN6 deficient clones.

Next, we assayed CCN6-deficient cells and controls for their growth ability in serum free medium and in the presence of IGF-1 in the condition media. In the complete absence of serum, CCN6-deficient cells were able to survive and were metabolically active when compared to controls (Fig. 4). Upon addition of IGF-1 we observed that inhibition of CCN6 enhanced the growth effects of IGF-1. Inhibition of CCN6 stimulated the growth of HME cells in serum free medium, however, when 25 ng of IGF-1 was added, we noted a 2.5-fold increase in cell growth, which did not increase significantly with increasing concentrations of recombinant IGF-1 in the medium.

**Figure 5:**
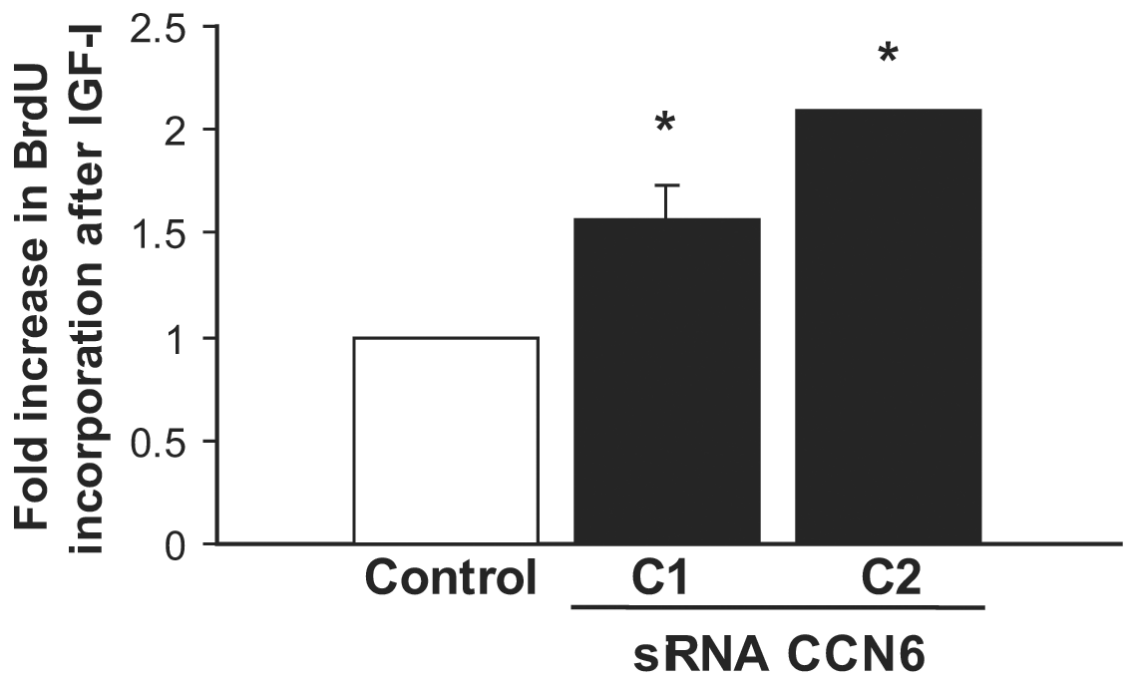
CCN6 inhibition enhances the proliferative effects of insulin-like growth factor-1 (IGF-1) on mammary epithelial cells. The bar graph shows that CCN6-deficient cells exhibited a significant increase in bromodeoxyuridine (BrdU) incorporation in response to treatment with IGF-1 (*t *test, *p *< 0.03 for both clones). The human mammary epithelial (HME) control cells did not exhibit differences in the S-phase fraction after the addition of IGF-1. The experiment was performed three times independently. Results are expressed as means ± SD for three independent experiments. C1 and C2 are two stable CCN6 deficient clones.

While IGF-1 stimulation did not affect the proliferative activity of the control cells, CCN6-deficient cells exhibited a significant increase in the S-phase fraction (Fig. 5). Taken together these data support the hypothesis that loss of CCN6 in HME cells results in loss of a growth regulatory mechanism which enhances the proliferative and growth response to IGF-1.

## Discussion

In this study we characterize the function of CCN6, a recently identified gene that is lost in inflammatory breast cancer, the most lethal form of breast cancer [6,25]. Here we show that inhibition of CCN6 expression induces the EMT of mammary epithelial cells and results in a highly motile and invasive phenotype. CCN6-deficient cells acquire anchorage-independent growth abilities and exhibit increased growth and proliferative response to IGF-1 through enhanced IGF-1R signaling.

To characterize the function of CCN6 in the breast epithelium, we first analyzed the phenotypic effects of stable inhibition of CCN6 expression by hairpin siRNA. Inhibition of CCN6 profoundly altered the phenotype of human mammary epithelial cells, leading to the acquisition of features of EMT. EMT involves the phenotypic alteration of epithelial cells to fibroblastoid, spindle, migratory, and more malignant cells, which show a mesenchymal gene expression program [32,33], and represents an important *in vitro *correlate of tumor progression [32,33]. Several proteins have been implicated in this process, which involves a dominant transcriptional repression that leads to a downregulation of E-cadherin expression and an increase in the expression of mesenchymal markers. The detailed mechanisms of EMT in breast epithelial cells remain elusive.

On the basis of our studies, inhibition of CCN6 results in a morphologic change towards a mesenchymal phenotype, which is accompanied by a marked downregulation of E-cadherin and cytokeratin, and an increase in vimentin, a mesenchymal marker. Functionally, CCN6 inhibition led to a pronounced increase in cell motility and invasion. The mechanism of E-cadherin downregulation brought about by the loss of CCN6 is intriguing. E-cadherin transcript expression was also inhibited in CCN6-deficient cells, suggesting that this effect is probably exerted at the transcriptional level, by epigenetic mechanisms such as hypermethylation and transcriptional silencing. These results were also obtained when CCN6 was knocked down in MCF10A cells (data not shown). It is possible that the loss of CCN6 might lead to increased methylation of the E-cadherin promoter, which has been shown to be correlated with a loss of E-cadherin expression in breast cancer cell lines and primary invasive ductal and lobular carcinomas of the breast [34,35]. It is also possible that CCN6 loss results in transcriptional silencing of E-cadherin, perhaps by the induction of transcriptional repressors, such as Snail [36,37] and SIP1/ZEB2 [36,37], known to inactivate the E-cadherin promoter. Supporting evidence has recently been reported by Sen and colleagues [38] showing that CCN6 is able to activate the SOX transcription factors by a similar mechanism.

CCN6 inhibition led to the acquisition of anchorage-independent growth abilities even under extremely low serum conditions. We therefore reasoned that loss of CCN6 might impair a central growth regulatory mechanism, rendering the cells more sensitive to the effect of even minimal amounts of growth factors such as EGF and IGF. Although CCN6 inhibition had no effect on the phosphorylation of the EGF receptor (data not shown), it markedly enhanced the growth effects of IGF-1, a potent mitogen for breast epithelial cells [39]. CCN6 inhibition led to an increase in the phosphorylation of IGF-1R and its main downstream target molecule IRS-1. This was coupled with an increase in the metabolic and proliferative activities of mammary epithelial cells. These data support a role for CCN6 a modulator of the growth effects of IGF-1 on the breast epithelium, and may have clinical implications, as CCN6 loss may mark an epithelium at increased risk of malignant transformation and might constitute a target for breast cancer prevention.

The IGF family of growth factors is crucial in the development and progression of breast cancer. Studies *in vitro *and *in vivo *have shown that IGFs promote the proliferation, survival, and metastatic ability of cancer cells [40-43]. The role of IGF-1R in promoting the proliferation of breast cancer cells is well established [39,44,45]. A role for IGF-1R in breast cancer metastases has been shown recently [40]. Compelling epidemiological and clinical data show that high concentrations of IGF-1 in serum are associated with increased mammographic density (one of the strongest predictors of breast cancer risk), and also reliably predict increased breast cancer risk specifically in premenopausal women [46]. High expression of IGF-1R has been demonstrated in most primary human breast cancers when compared with normal or benign breast tissue, and hyperactivation of IGF-1R in breast cancer has been linked with increased radioresistance and cancer recurrence at the primary site [39,44,45]. High levels of IRS-1, a major signaling molecule downstream of the IGF-1R, are correlated with tumor size and shorter disease-free survival in estrogen receptor-positive breast cancer patients [47,48].

The effect of CCN6 inhibition on the phosphorylation of the IGF-1R and IRS-1 is consistent with a growth regulatory effect on the mammary epithelium. The relationship between the IGF family and CCN6 emerges from our previous investigations as well as from another laboratory [14,27,38]. CCN6-rich medium induces a decrease in IGF-1-stimulated IGF-1R phosphorylation in breast cancer cells, and then results in an inhibition of cellular proliferation [27]. In another system, Sen and colleagues [38] reported that anti-IGF-1 neutralizing antibody partly blocks the CCN6-mediated upregulation of COL2A1 mRNA in immortalized chondrocyte cell lines. In view of these data, we postulate that CCN6 may modulate the availability of IGF-1 to the IGF-1R, conceivably by binding and sequestering IGF-1, and/or by interfering with other regulators of the IGF signaling pathway.

## Conclusion

We propose a model whereby CCN6 serves as an important growth regulatory protein of the mammary epithelium and controls the cellular responses to the growth stimulatory effects of IGF-1. Loss of CCN6 expression alters the phenotype of the breast epithelium and promotes motility and invasion. The CCN6-deficient cells are extremely sensitive to the growth stimulatory effects of IGF-1. The uncontrolled growth may in turn predispose to neoplasia. It is possible that pharmacological restoration of the CCN6 autocrine regulatory loop could be targeted to prevent malignant transformation of mammary epithelial cells.

## Abbreviations

bp = base pair; EGF = epidermal growth factor; EMT = epithelial-mesenchymal transition; FBS = fetal bovine serum; HME = human mammary epithelial; IGF = insulin-like growth factor; IGF-1R = insulin-like growth factor-1 receptor; MTT = 3-(4,5-dimethylthiazol-2-yl)-2,5-diphenyl-2*H*-tetrazolium bromide; RT-PCR = reverse transcriptase-mediated polymerase chain reaction; siRNA = short interfering RNA.

## Competing interests

The authors declare that they have no competing interests.

## Authors' contributions

YZ carried out the cell culture, transfections, western blotting, RT-PCR, functional assays, and IGF-1 assays. She drafted the manuscript. QP assisted with the optimization of techniques, and was involved in the overall planning of experiments. HZ contributed her knowledge on flow cytometry and performed the BrdU incorporation assays. She assisted with the planning and interpretation of IGF-1 experiments, and manuscript writing. SDM conceived some ideas on the study, and contributed to the experimental plan, especially on the IGF-1 connection. She assisted in trouble shooting experiments. She helped planning the figures and edited the manuscript. CGK contributed ideas for the study and participated in the design and coordination of the study. She wrote and edited the manuscript significantly. She was involved and directed all the aspects of the study. SDM and CGK share senior authorship. All authors read and approved the final manuscript.
